# Poly[[diaquadi-μ_2_-cyanido-bis­(μ_2_-pyrazine-2-carboxyl­ato)dicopper(I)copper(II)] dihydrate]

**DOI:** 10.1107/S1600536811019453

**Published:** 2011-06-04

**Authors:** Guang Fan, Jia-juan Sun, Min-yan Zheng, San-ping Chen, Sheng-Li Gao

**Affiliations:** a College of Chemistry & Chemical Engineering, Xianyang Normal University, Xianyang 712000, Shaanxi, People’s Republic of China; b College of Chemistry & Materials Science, Northwest University, Xi’an 710069, Shaanxi, People’s Republic of China

## Abstract

In the title compound, {[Cu^II^Cu^I^_2_(C_5_H_3_N_2_O_2_)_2_(CN)_2_(H_2_O)_2_]·2H_2_O}_*n*_, the Cu^II^ atom lies on an inversion centre and is octa­hedrally coordinated by two N atoms and two O atoms from opposing pyrazine-2-carboxyl­ate (2-pac) ligands and two water O atoms. The Cu^I^ atom has a triangular geometry, coordinated by one N atom and one C atom from two bridging cyanide ligands, and another N atom from the 2-pac ligand. The three-dimensional structure features a succession of two-dimensional sheets containing [Cu(CN)]_*n*_ chains linked by Cu(2-pac)_2_(H_2_O)_2_ groups. The coordinated and free water mol­ecules are involved in an extended three-dimensional hydrogen-bond network with the 2-pac ligands.

## Related literature

For applications of metal-organic frameworks (MOFs), see: Klein *et al.* (1982 ▶); Li *et al.* (2004 ▶); Plater *et al.* (2001 ▶); Thomas (1978 ▶). For a related structure, see: Fan *et al.* (2006 ▶). 
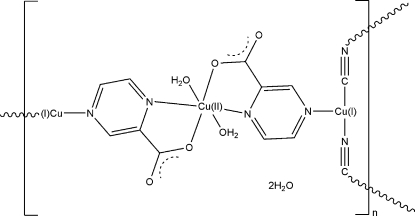


## Experimental

### 

#### Crystal data

[Cu_3_(C_5_H_3_N_2_O_2_)_2_(CN)_2_(H_2_O)_2_]·2H_2_O
*M* *_r_* = 560.91
Monoclinic, 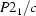

*a* = 13.8297 (4) Å
*b* = 9.4906 (3) Å
*c* = 7.1272 (3) Å
β = 100.768 (3)°
*V* = 918.99 (6) Å^3^
*Z* = 2
Mo *K*α radiation
μ = 3.50 mm^−1^
*T* = 296 K
0.10 × 0.08 × 0.05 mm

#### Data collection

Bruker SMART APEXII CCD diffractometer
Absorption correction: multi-scan (*SADABS*; Sheldrick, 1996) ▶ *T*_min_ = 0.721, *T*_max_ = 0.845
4141 measured reflections
1615 independent reflections
1269 reflections with *I* > 2σ(*I*)
*R*_int_ = 0.022

#### Refinement

*R*[*F* ^2^ > 2σ(*F* ^2^)] = 0.029
*wR*(*F* ^2^) = 0.095
*S* = 0.99
1615 reflections
145 parameters
6 restraints
H atoms treated by a mixture of independent and constrained refinement
Δρ_max_ = 0.36 e Å^−3^
Δρ_min_ = −0.36 e Å^−3^

### 

Data collection: *APEX2* (Bruker, 2004 ▶); cell refinement: *SAINT* (Bruker, 2004 ▶); data reduction: *SAINT*; program(s) used to solve structure: *SHELXS97* (Sheldrick, 2008 ▶); program(s) used to refine structure: *SHELXL97* (Sheldrick, 2008 ▶); molecular graphics: *SHELXTL/PC* (Sheldrick, 2008 ▶); software used to prepare material for publication: *SHELXTL/PC*.

## Supplementary Material

Crystal structure: contains datablock(s) I, global. DOI: 10.1107/S1600536811019453/vn2007sup1.cif

Structure factors: contains datablock(s) I. DOI: 10.1107/S1600536811019453/vn2007Isup2.hkl

Additional supplementary materials:  crystallographic information; 3D view; checkCIF report

## Figures and Tables

**Table 1:** Selected bond lengths (Å)

Cu1—O1	1.978 (2)
Cu1—N1	2.003 (3)
Cu1—O3	2.378 (3)
Cu2—C6	1.865 (3)
Cu2—N3^i^	1.886 (4)
Cu2—N2	2.163 (3)

Symmetry code: (i) 
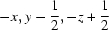
.

**Table 2:** Hydrogen-bond geometry (Å, °)

*D*—H⋯*A*	*D*—H	H⋯*A*	*D*⋯*A*	*D*—H⋯*A*
O4—H4*A*⋯O2	0.86 (2)	2.08 (3)	2.910 (5)	160 (7)
O3—H3*B*⋯O1^ii^	0.82 (2)	2.11 (2)	2.883 (3)	156 (4)
O3—H3*A*⋯O2^iii^	0.84 (2)	1.94 (2)	2.783 (4)	172 (4)

Symmetry codes: (ii) 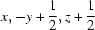; (iii) 
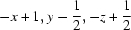
.

**Fig. 1.**
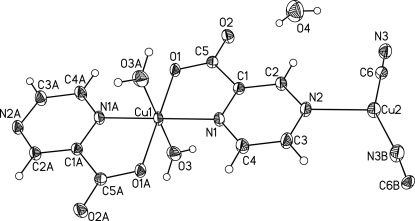
A view of the molecular structure of (I) with the atom-labeling scheme. Displacement ellipsoids are drawn at the 30% probability level and H atoms are shown as small spheres of arbitrary radii. Symmetry code for A: 1-x, -y, -z; B: –x, -0.5+y, 0.5-z.

**Fig. 2.**
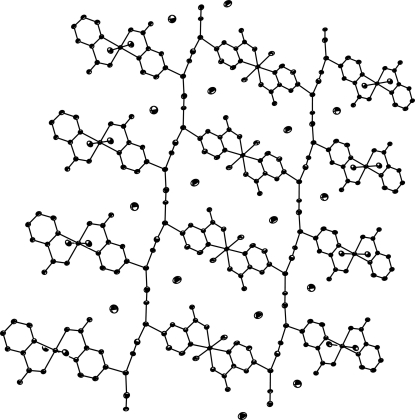
Two-dimensional sheet structure for complex (I). Hydrogen atoms have been omitted for clarity.

**Fig. 3.**
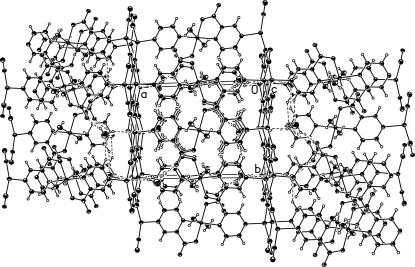
Three-dimensional stacking diagram for complex (I) along the c axis.

## supplementary crystallographic information

### Comment

Single crystal diffraction has revealed that complex (I) crystallizes in the monoclinic space group *P*2_1_/*c* featuring two-dimensional networks through chain-like [Cu(CN)]_n_ units linked by Cu(2-pac)_2_(H_2_O)_2_. As shown in Fig. 1, there are two crystallographic inequivalent copper atoms. The Cu(1) atom is divalent and Cu(2) is monovalent. Cu(1) adopts a distorted octahedral geometry by two N and two O atoms from the 2-pac ligands in the equatorial plane whereas the axial positions are occupied by two water molecules with 1.9781 (17)Å for Cu1—O1; 2.002 (2)Å for Cu1—N1; 2.371 (2)Å for Cu1—O3. Each Cu(2) atom has a triangular geometry, coordinated to one N atom and one C atom from two bridging cyanide ligands and another N atom from Cu(2-pac)_2_(H_2_O)_2_, with 1.860 (3)Å for Cu2—C6; 1.885 (3)Å for Cu2—N3; 2.163 (2)Å for Cu2—N2.

Fig. 2 shows the independent cyanide ligands bridging Cu(2) to form a zigzag chain of [Cu(CN)]_n_ units. Such chains are interconnected through two Cu(2-pac)_2_(H_2_O)_2_ N donor ligands giving rise to a two-dimensional sheet network. Furthermore, an extensive hydrogen bonding network is formed, which involves the coordinated, free water molecules and the 2-pac ligand substituents, affording a three-dimensional network, as shown in Fig. 3. It is noted that one proton of the free water molecule has no apparent hydrogen acceptor atom.

The present structure is quite different from the mixed-valence copper complex [Cu^II^Cu^I^(2-pac)_2_(NO_3_)(H_2_O)]_n_ reported by Fan *et al.* (2006). In the latter structure the coordination number of monovalent Cu atom is 4,  but for the present structure the coordination number is 3.

### Experimental

Red crystals from complex (I) were obtained by hydrothermal synthesis of a mixture of Cu(NO_3_)_2_.3H_2_O (0.1241 g, 0.5 mmol), 0.4 ml H_3_PO_3_ and 2-pac (0.0673 g, 0.5 mmol) in 6 ml H_2_O, sealed in a Teflon-lined stainless container, heated at 363 K for 24 h and slowly cooled to room temperature.

### Refinement

All H atoms attached to C atoms from the organic ligands were generated in idealized positions and constrained to ride on their parent atoms, with d(C—H) = 0.93 Å, *U*_iso_=1.2*U*_eq_ (C). The water H-atoms were located in a different Fourier map, and the geometry of the two water molecules was restrained to its ideal geometry by in total six restraints on angles and bond distances.

### Crystal data

**Table:**

[Cu_3_(C_5_H_3_N_2_O_2_)_2_(CN)_2_(H_2_O)_2_]·2H_2_O	*F*(000) = 558
*M**_r_* = 560.91	*D*_x_ = 2.027 Mg m^−^^3^
Monoclinic, *P*2_1_/*c*	Mo *K*α radiation, λ = 0.71073 Å
Hall symbol: -P 2ybc	Cell parameters from 117 reflections
*a* = 13.8297 (4) Å	θ = 2.5–19.1°
*b* = 9.4906 (3) Å	µ = 3.50 mm^−^^1^
*c* = 7.1272 (3) Å	*T* = 296 K
β = 100.768 (3)°	Block, red
*V* = 918.99 (6) Å^3^	0.10 × 0.08 × 0.05 mm
*Z* = 2	

### Data collection

**Table:**

Bruker SMART APEXII CCD diffractometer	1615 independent reflections
Radiation source: fine-focus sealed tube	1269 reflections with *I* > 2σ(*I*)
graphite	*R*_int_ = 0.022
φ and ω scans	θ_max_ = 25.1°, θ_min_ = 3.6°
Absorption correction: multi-scan (*SADABS*; Sheldrick, 1996)	*h* = −16→16
*T*_min_ = 0.721, *T*_max_ = 0.845	*k* = −11→11
4141 measured reflections	*l* = −7→8

### Refinement

**Table:**

Refinement on *F*^2^	Primary atom site location: structure-invariant direct methods
Least-squares matrix: full	Secondary atom site location: difference Fourier map
*R*[*F*^2^ > 2σ(*F*^2^)] = 0.029	Hydrogen site location: inferred from neighbouring sites
*wR*(*F*^2^) = 0.095	H atoms treated by a mixture of independent and constrained refinement
*S* = 0.99	*w* = 1/[σ^2^(*F*_o_^2^) + (0.0484*P*)^2^ + 1.5206*P*] where *P* = (*F*_o_^2^ + 2*F*_c_^2^)/3
1615 reflections	(Δ/σ)_max_ < 0.001
145 parameters	Δρ_max_ = 0.36 e Å^−^^3^
6 restraints	Δρ_min_ = −0.36 e Å^−^^3^

### Special details

**Table:**

Geometry. All e.s.d.'s (except the e.s.d. in the dihedral angle between two l.s. planes) are estimated using the full covariance matrix. The cell e.s.d.'s are taken into account individually in the estimation of e.s.d.'s in distances, angles and torsion angles; correlations between e.s.d.'s in cell parameters are only used when they are defined by crystal symmetry. An approximate (isotropic) treatment of cell e.s.d.'s is used for estimating e.s.d.'s involving l.s. planes.
Refinement. Refinement of *F*^2^ against ALL reflections. The weighted *R*-factor *wR* and goodness of fit *S* are based on *F*^2^, conventional *R*-factors *R* are based on *F*, with *F* set to zero for negative *F*^2^. The threshold expression of *F*^2^ > σ(*F*^2^) is used only for calculating *R*-factors(gt) *etc*. and is not relevant to the choice of reflections for refinement. *R*-factors based on *F*^2^ are statistically about twice as large as those based on *F*, and *R*- factors based on ALL data will be even larger.

### Fractional atomic coordinates and isotropic or equivalent isotropic displacement parameters (Å^2^)

**Table:**

	*x*	*y*	*z*	*U*_iso_*/*U*_eq_	
Cu1	0.5000	0.0000	0.0000	0.0296 (2)	
Cu2	0.03571 (3)	0.00934 (4)	0.21527 (8)	0.0366 (2)	
O1	0.45839 (17)	0.1989 (2)	−0.0394 (4)	0.0316 (6)	
O2	0.33417 (17)	0.3429 (2)	−0.0167 (4)	0.0356 (6)	
O3	0.5540 (2)	0.0613 (3)	0.3263 (4)	0.0433 (7)	
H3A	0.588 (3)	−0.001 (4)	0.393 (6)	0.065*	
H3B	0.524 (3)	0.113 (4)	0.390 (6)	0.065*	
C5	0.3724 (2)	0.2253 (3)	−0.0104 (5)	0.0258 (7)	
N1	0.3627 (2)	−0.0237 (3)	0.0479 (5)	0.0297 (7)	
N2	0.1722 (2)	−0.0069 (3)	0.1101 (5)	0.0330 (7)	
N3	−0.0012 (2)	0.3197 (4)	0.2534 (5)	0.0422 (8)	
C1	0.3146 (2)	0.0996 (3)	0.0357 (5)	0.0253 (7)	
C4	0.3148 (3)	−0.1381 (4)	0.0874 (6)	0.0401 (10)	
H4	0.3458	−0.2254	0.0941	0.048*	
C2	0.2195 (2)	0.1067 (3)	0.0676 (5)	0.0293 (8)	
H2	0.1877	0.1935	0.0591	0.035*	
C6	0.0122 (2)	0.2013 (3)	0.2401 (5)	0.0288 (8)	
C3	0.2199 (3)	−0.1284 (4)	0.1185 (6)	0.0405 (10)	
H3	0.1881	−0.2100	0.1463	0.049*	
O4	0.1922 (3)	0.5271 (4)	0.1144 (8)	0.0884 (13)	
H4A	0.229 (4)	0.457 (5)	0.093 (11)	0.133*	
H4B	0.133 (2)	0.495 (7)	0.108 (13)	0.133*	

### Atomic displacement parameters (Å^2^)

**Table:**

	*U*^11^	*U*^22^	*U*^33^	*U*^12^	*U*^13^	*U*^23^
Cu1	0.0222 (3)	0.0210 (3)	0.0499 (4)	0.0014 (2)	0.0178 (3)	0.0025 (2)
Cu2	0.0345 (3)	0.0214 (3)	0.0572 (4)	−0.00078 (17)	0.0176 (2)	−0.0008 (2)
O1	0.0256 (12)	0.0243 (12)	0.0487 (16)	−0.0007 (10)	0.0170 (11)	0.0041 (11)
O2	0.0297 (12)	0.0217 (13)	0.0564 (18)	0.0009 (10)	0.0109 (12)	0.0014 (11)
O3	0.0502 (17)	0.0366 (16)	0.0452 (18)	0.0128 (13)	0.0138 (14)	−0.0037 (13)
C5	0.0267 (17)	0.0226 (17)	0.029 (2)	−0.0009 (13)	0.0070 (15)	−0.0010 (14)
N1	0.0240 (14)	0.0234 (15)	0.0449 (19)	0.0010 (12)	0.0147 (14)	0.0007 (13)
N2	0.0267 (15)	0.0260 (16)	0.050 (2)	−0.0008 (11)	0.0173 (15)	0.0018 (13)
N3	0.0386 (17)	0.042 (2)	0.050 (2)	0.0022 (15)	0.0172 (16)	0.0012 (16)
C1	0.0251 (16)	0.0212 (17)	0.031 (2)	0.0003 (13)	0.0079 (14)	−0.0012 (14)
C4	0.036 (2)	0.0215 (18)	0.068 (3)	0.0060 (15)	0.023 (2)	0.0058 (18)
C2	0.0248 (17)	0.0225 (18)	0.042 (2)	−0.0004 (14)	0.0106 (16)	−0.0012 (15)
C6	0.0322 (18)	0.0183 (17)	0.039 (2)	0.0033 (14)	0.0139 (16)	−0.0011 (15)
C3	0.0326 (19)	0.0267 (19)	0.068 (3)	−0.0008 (16)	0.0245 (19)	0.0055 (19)
O4	0.082 (3)	0.077 (3)	0.115 (4)	0.013 (2)	0.043 (3)	−0.001 (3)

### Geometric parameters (Å, °)

**Table:**

Cu1—O1^i^	1.978 (2)	C5—C1	1.507 (4)
Cu1—O1	1.978 (2)	N1—C4	1.329 (4)
Cu1—N1^i^	2.003 (3)	N1—C1	1.340 (4)
Cu1—N1	2.003 (3)	N2—C3	1.324 (5)
Cu1—O3^i^	2.378 (3)	N2—C2	1.326 (4)
Cu1—O3	2.378 (3)	N3—C6	1.146 (5)
Cu2—C6	1.865 (3)	N3—Cu2^iv^	1.886 (4)
Cu2—N3^ii^	1.886 (4)	C1—C2	1.377 (5)
Cu2—N2	2.163 (3)	C4—C3	1.375 (5)
Cu2—Cu2^iii^	3.0481 (11)	C4—H4	0.9300
O1—C5	1.269 (4)	C2—H2	0.9300
O2—C5	1.232 (4)	C3—H3	0.9300
O3—H3A	0.844 (19)	O4—H4A	0.86 (2)
O3—H3B	0.824 (19)	O4—H4B	0.87 (2)
			
O1^i^—Cu1—O1	180.00 (14)	O2—C5—O1	125.6 (3)
O1^i^—Cu1—N1^i^	82.62 (10)	O2—C5—C1	118.9 (3)
O1—Cu1—N1^i^	97.38 (10)	O1—C5—C1	115.5 (3)
O1^i^—Cu1—N1	97.38 (10)	C4—N1—C1	117.8 (3)
O1—Cu1—N1	82.62 (10)	C4—N1—Cu1	130.8 (2)
N1^i^—Cu1—N1	180.00 (3)	C1—N1—Cu1	111.5 (2)
O1^i^—Cu1—O3^i^	86.28 (10)	C3—N2—C2	117.1 (3)
O1—Cu1—O3^i^	93.72 (10)	C3—N2—Cu2	120.4 (2)
N1^i^—Cu1—O3^i^	89.72 (11)	C2—N2—Cu2	121.4 (2)
N1—Cu1—O3^i^	90.28 (11)	C6—N3—Cu2^iv^	173.8 (3)
O1^i^—Cu1—O3	93.72 (10)	N1—C1—C2	120.7 (3)
O1—Cu1—O3	86.28 (10)	N1—C1—C5	115.4 (3)
N1^i^—Cu1—O3	90.28 (11)	C2—C1—C5	124.0 (3)
N1—Cu1—O3	89.72 (11)	N1—C4—C3	120.6 (3)
O3^i^—Cu1—O3	180.00 (14)	N1—C4—H4	119.7
C6—Cu2—N3^ii^	150.28 (16)	C3—C4—H4	119.7
C6—Cu2—N2	106.34 (13)	N2—C2—C1	121.7 (3)
N3^ii^—Cu2—N2	103.29 (12)	N2—C2—H2	119.2
C6—Cu2—Cu2^iii^	97.07 (12)	C1—C2—H2	119.2
N3^ii^—Cu2—Cu2^iii^	91.27 (11)	N3—C6—Cu2	178.9 (4)
N2—Cu2—Cu2^iii^	77.67 (9)	N2—C3—C4	122.2 (3)
C5—O1—Cu1	114.9 (2)	N2—C3—H3	118.9
Cu1—O3—H3A	115 (3)	C4—C3—H3	118.9
Cu1—O3—H3B	126 (3)	H4A—O4—H4B	107 (4)
H3A—O3—H3B	113 (3)		

Symmetry codes: (i) −*x*+1, −*y*, −*z*; (ii) −*x*, *y*−1/2, −*z*+1/2; (iii) −*x*, −*y*, −*z*; (iv) −*x*, *y*+1/2, −*z*+1/2.

### Hydrogen-bond geometry (Å, °)

**Table:**

*D*—H···*A*	*D*—H	H···*A*	*D*···*A*	*D*—H···*A*
O4—H4A···O2	0.86 (2)	2.08 (3)	2.910 (5)	160 (7)
O3—H3B···O1^v^	0.82 (2)	2.11 (2)	2.883 (3)	156 (4)
O3—H3A···O2^vi^	0.84 (2)	1.94 (2)	2.783 (4)	172 (4)

Symmetry codes: (v) *x*, −*y*+1/2, *z*+1/2; (vi) −*x*+1, *y*−1/2, −*z*+1/2.
